# Editorial: Cognitive NeuroIntelligence

**DOI:** 10.3389/fncom.2021.718518

**Published:** 2021-07-26

**Authors:** Yiying Song, Si Wu, Ke Zhou, Jia Liu

**Affiliations:** ^1^Faculty of Psychology, Beijing Normal University, Beijing, China; ^2^School of Electronics Engineering and Computer Science, Peking University, Beijing, China; ^3^Tsinghua Laboratory of Brain and Intelligence, Tsinghua University, Beijing, China

**Keywords:** artificial neural network, cognitive neuroscience, cognitive neurointelligence, cognitive-neuroscience-inspired AI, AI-inspired cognitive neuroscience

In recent years, fascinating progresses have been made in utilizing artificial intelligence to solve a broad range of problems. AI systems today can match and even outperform human performance in certain challenging tasks, including visual cognition. Recent AI advances in deep learning have been largely inspired by neuroscience research on biological brain and guided by architectural constrains from biological neural networks. In this Research Topic, we advocate further interactions between the fields of AI and cognitive neuroscience to benefit both fields. The topic of Cognitive Neurointelligence, starting from May 25th, 2020 and ending on November 20th, 2020, was organized by Jia Liu, Si Wu, Ke Zhou, and Yiying Song.

On one hand, there is great potential for cognitive neuroscience to benefit AI (cognitive-neuroscience-inspired AI). The structures and functions of neural systems, which result from hundreds of millions of years evolution, are optimized for animals processing information in order to survive in natural environments. They naturally serve as the resources inspiring us to develop AI. In addition to this, cognitive neuroscience can also benefit AI research from a new aspect. The end-to-end learning strategy makes deep convolutional neural networks (DCNNs) remaining to be “black boxes,” where the algorithms and computations of the networks are poorly understood. Techniques and approaches available in cognitive neuroscience, including experimental paradigms, data analysis techniques, and theoretical hypotheses, can serve as a repertoire of tools for unveiling the black boxes of DCNNs, illuminating the algorithms and computations inside the networks.

On the other hand, AI can make fundamental contributions to cognitive neuroscience as well (AI-inspired cognitive neuroscience). In addition to serving as advanced mathematical tools for analyzing big data in neuroscience, models of AI can also give us insight into understanding the inner mechanisms of biological brain and intelligence, for instance, DCNNs have offered the best models of biological visual systems to date. More importantly, biological brains are the result of evolution; and analogically we can manipulate loss functions, architectures, and datasets of DCNNs to “re-run” evolution and therefore to pry open secrets that lead to the emergence of the human brain and mind.

Therefore, the purpose of this Research Topic is to bring together research efforts from AI and cognitive neuroscience, seeking to integrate AI and cognitive neuroscience toward a new field of cognitive neurointelligence.

In the direction of cognitive-neuroscience-inspired AI, six papers in this special issue aimed to develop advanced information processing techniques inspired by biological systems. Motived by the unsupervised learning behavior of humans, Ji et al. proposed an unsupervised few-shot learning algorithm for object classification. Motivated by the rapid topology perception of humans, Wang et al. proposed a gap-junction network for fast topology detection in images. Inspired by the balance of excitation and inhibition (E-I) interactions in neural systems, Tian G. et al. proposed an E-I balanced network for fast signal detection. Based on the biologically plausible global feedback and the local STDP learning rule, Zhao et al. proposed a new method to train multi-layer spiking neural networks. Applying biological learning rules, Fang et al. developed spiking neural networks for sequence generation. Zhou et al. revealed that the function connectivity of the brain network accounts for critical dynamics, and the latter leads to efficient information processing. In addition, three papers in this special issue applied methods in cognitive neuroscience to understand inner representations in DCNNs. Liu et al. applied the concepts and measures of coding schemes from neuroscience studies to DCNNs and provided evidence that DCNNs adopted a hierarchically-evolved sparse coding scheme to represent objects as the biological brain does. Song et al. adopted a reverse-correlation approach in psychophysical studies and found that both DCNNs and humans utilized similar inner representations to perform the task of face gender classification. Tian J. et al. explored the phenomenon and mechanism of biased behaviors in DCNNs by borrowing the paradigms and theories from a classical race bias (i.e., the other race effect) in humans and found a human-like multidimensional face representation in DCNN. Together, these studies suggest the possibility that DCNNs and humans may use an implementation-independent representation to achieve the same computation goal.

In the direction of AI-inspired cognitive neuroscience, two studies in this issue used DCNNs as a model to inform our understanding of human cognition. Unlike studies on humans where perceptual experiences are always intermingled with conceptual guidance, Huang et al. used DCNNs to provide evidence that the semantic relatedness of object categories can automatically emerge from perceptual experiences without top-down conceptual guidance. In addition, investigation on the role of nature vs. nurture in the formation of domain-specific modules in biological brains cannot easily dissociate the effects of visual experience from genetic predisposition. To overcome this limitation, Xu et al. built a model of selective deprivation of the experience on faces with a DCNN and demonstrated that domain-specificity may evolve from non-specific experience without genetic predisposition, and is further fine-tuned by domain-specific experience. In other two studies, Zhang et al. applied AI algorithms to improve target detection in neural signals, and Zheng et al. applied DCNNs to investigate the transfer learning effects based on local and global invariant features.

Finally, to meet the objective of crosstalk between the AI and cognitive neuroscience, Chen et al. presented a Python-based toolbox, DNN Brain, which enables researchers from both fields to conveniently map the internal representations of DNNs and brain, and examine their correspondences.

## Author Contributions

All authors listed have made a substantial, direct and intellectual contribution to the work, and approved it for publication.

## Conflict of Interest

The authors declare that the research was conducted in the absence of any commercial or financial relationships that could be construed as a potential conflict of interest.

## Publisher's Note

All claims expressed in this article are solely those of the authors and do not necessarily represent those of their affiliated organizations, or those of the publisher, the editors and the reviewers. Any product that may be evaluated in this article, or claim that may be made by its manufacturer, is not guaranteed or endorsed by the publisher.

